# Dihydroartemisinin Induces Endothelial Cell Autophagy through Suppression of the Akt/mTOR Pathway

**DOI:** 10.7150/jca.33704

**Published:** 2019-10-15

**Authors:** Jing Liu, Yanjun Ren, Yinglong Hou, Caiqing Zhang, Bei Wang, Xiaorui Li, Rong Sun, Ju Liu

**Affiliations:** 1 Laboratory of Microvascular Medicine, Medical Research Center, Shandong Provincial Qianfoshan Hospital, the First Hospital Affiliated with Shandong First Medical University, Jinan, Shandong, PR China;; 2 Department of Orthopaedics; Shandong Provincial Qianfoshan Hospital, the First Hospital Affiliated with Shandong First Medical University, Jinan, Shandong, PR China; 3 Department of Cardiology; Shandong Provincial Qianfoshan Hospital, the First Hospital Affiliated with Shandong First Medical University, Jinan, Shandong, PR China; 4 Department of Respiratory Medicine; Shandong Provincial Qianfoshan Hospital, the First Hospital Affiliated with Shandong First Medical University, Jinan, Shandong, PR China; 5 Department of Ultrasound, Shandong Provincial Qianfoshan Hospital, the First Hospital Affiliated with Shandong First Medical University, Jinan, Shandong, PR China;; 6 Graduate School, Shandong First Medical University & Shandong Academy of Medical Sciences, Jinan, China;; 7 Advanced Medical Research Institute, Shandong University, Jinan, China;; 8 The Second Hospital of Shandong University, Jinan, China.

**Keywords:** Dihydroartemisinin, Autophagy, HUVEC, Akt, MTOR

## Abstract

**Aims:** Dihydroartemisinin (DHA), a derivative of artemisinin, suppresses angiogenesis by regulating endothelial cell phenotypes. In this study, we investigated the effect of DHA on endothelial cell autophagy and the underlying mechanisms.

**Methods:** Human umbilical vein endothelial cells (HUVECs) were treated with DHA. Formation of autophagosomes in HUVECs was observed by fluorescence microscope after pcDNA3.1-green fluorescent protein (GFP)-microtubule-associated protein 1 light chain 3 (LC3) plasmids transfection. Dichlorofluorescein diacetate (DCFH-DA) staining was used to detect intracellular reactive oxygen species (ROS). Western blot was performed to detect the protein levels of LC3, p62, beclin 1, autophagy-related protein (Atg) 5, p-Akt (protein kinase B), p-mTOR (mammalian target of rapamycin), p-4E-BP1 (eukaryotic translation initiation factor 4E-binding protein 1), and p-p70S6K (p70 ribosomal S6 kinase).

**Results:** DHA increased LC3-II and the number of fluorescent GFP-LC3 puncta in HUVECs. Silencing ATG5 by siRNA interference attenuated DHA-induced LC3-II elevation. DHA enhanced ROS production, but pretreatment with antioxidant N-acety-l-cysteine (NAC) failed to reduce DHA-induced autophagy in HUVECs. Pretreatment with PD98059, SP600125 and SB203580, the inhibitors of ERK, JNK, and p38 MAPK, did not reverse autophagy in DHA-treated HUVECs. DHA significantly reduced phosphorylation of Akt, mTOR, p70S6K, 4E-BP1 in HUVECs. Rapamycin, an mTOR antagonist, compromised DHA-induced autophagy.

**Conclusion:** DHA induces autophagy in HUVECs by inhibition of the Akt/mTOR pathway

## Introduction

Angiogenesis, the formation of new blood vessels, is triggered by elevated proangiogenic factors in the microenvironment 1. It requires endothelial cells (ECs) activation, migration, proliferation and tube formation 1. The newly formed blood vessels supply nutrition and oxygen for tissues and thereby are essential for development, wound healing, tumor growth and metastasis 2-4. Inhibiting angiogenesis has become a promising option for anti-tumor therapies 5.

Autophagy is an evolutionarily conservative process for cells to clear dysfunctional organelles or misfolded proteins 6. The autophagosomes engulf and transport the cargos to fuse with lysosome. Within the lysosome, the contents of the autophagosome are degraded via acidic lysosomal hydrolases 6. Autophagy is induced by stresses such as starvation, hypoxia, oxidative stress and endoplasmic reticulum stress 7 and requires activation of Atg 8. Autophagy is closely related to angiogenesis. Angiogenic factor with G patch and FHA domains 1 (AGGF1)-induced therapeutic angiogenesis requires the induction of autophagy in ECs 9. Long non-coding (lnc) RNA Wilms tumor 1 associated protein pseudogene 1 (WTAPP1) enhances migration and angiogenesis of endothelial progenitor cells through activation of autophagy 10.

DHA, a semi-synthetic derivative of artemisinin, is widely used as an anti-malarial drug. We and others have reported a potent anti-angiogenetic function of DHA 11-13. DHA inhibits growth, migration, proliferation and tube formation of ECs 14, 15, all of which are necessary for angiogenesis. DHA also downregulates the expression of vascular endothelial growth factor receptor (VEGFR) 2 in HUVECs through inhibiting the binding activity of nuclear factor (NF)-κB to VEGFR2 promoter 12. However, the effect of DHA on endothelial cell autophagy has not been reported.

In the present study, we investigated the effects of DHA on autophagy in HUVECs and explored the underlying mechanisms. We demonstrated that DHA induces autophagy by inhibition of the Akt/mTOR signaling pathway, not through regulation of ROS or MAPK signaling.

## Materials and Methods

### Regents

DHA was purchased from Sigma-Aldrich (St. Louise, MO, USA). NAC and ROS detection assay kit were purchased from Beyotime Biotechnology (Shanghai, China). Antibodies against p62, beclin1, LC3, mTOR, p-mTOR (Ser2448), p-p70S6K (Thr389), p-4EBP1 (Thr37/46), p70S6K and 4EBP1 were purchased from Cell Signaling Technology (Danvers, MA, USA). Antibodies against p-AKT (Ser473) and AKT were purchased from Abcam (Cambridge, CA, USA). Horseradish peroxidases (HRP)-conjugated secondary antibodies were purchased from Sigma-Aldrich (St. Louise, MO, USA).

### Cell culture

HUVECs were purchased from American Type Culture Collection (Manassas, VA, USA). HUVECs were cultured in Dulbecco's modified Eagle's medium with 10% (v/v) fetal bovine serum and antibiotics (100 IU/ml penicillin and 100 mg/ml streptomycin) at 37 ℃ with a humidified atmosphere of 5% CO_2_/ 21% O_2_.

### Western blot

Cells were harvested and washed twice with PBS and then lysed in RIPA buffer with protease and phosphatase inhibitors on ice. After heat-denature, equal amounts of protein were separated by sodium dodecyl sulfate-polyacrylamide gel electrophoresis (SDS-PAGE) and transferred to polyvinylidene fluoride (PVDF) membranes. The membranes were blocked with 5% skim milk for 1 h and incubated with primary antibodies at 4 ℃ overnight. After washed with TBST for 3 times, the membranes were incubated with appropriate HRP-conjugated secondary antibodies. Enhanced chemiluminescence system (Millipore, Billerica, MA, USA) was used to detect immunoreactivity. GAPDH and β-actin were used as loading controls.

### Transfection of pcDNA3.1-GFP-LC3 plasmids

HUVECs at 90% confluence were transfected with 4 μg pcDNA3.1-GFP-LC3 plasmids (Biovector Science Lab, Beijing, China) by use of Lipofectamine^TM^ 2000 (Invitrogen, Carlsbad, CA) following the manufacturer's instructions. After transfection for 24 h, cells were exposed to 35 μM DHA for another 24 h. LC3 fluorescent puncta were detected by fluorescence microscope.

### *ATG5* siRNA transfection

HUVECs at 50% confluence were transiently transfected with *ATG5* siRNA (GenePharma, Suzhou, China) by using Lipofectamine^TM^ 2000 (Invitrogen, Carlsbad, CA). After transfection for 48 h, the medium was replaced with normal DMEM medium, and cells were treated with DHA (35 μM) for 24 h. Scramble siRNA was used as a negative control. The effect of gene silencing was estimated by Western blot.

### Intracellular ROS detection

Following DHA treatment, intracellular ROS levels were detected by fluorescence microscope after DCFH-DA staining. Briefly, the HUVECs were incubated with 10 μM DCFH-DA for 20 min at 37 ℃ in the dark, and were washed with serum-free medium for three times. The fluorescence was excited at the wavelength of 488 nm and the corresponding emission wavelength was 525 nm.

### Statistical analysis

All data were presented as mean ± SEM. Student *t* test or one-way ANOVA analysis was performed using GraphPad Prism 5. *P* value <0.05 was considered to indicate a statistically significant difference.

## Results

### DHA induces autophagy in HUVECs

**Figure 1:**
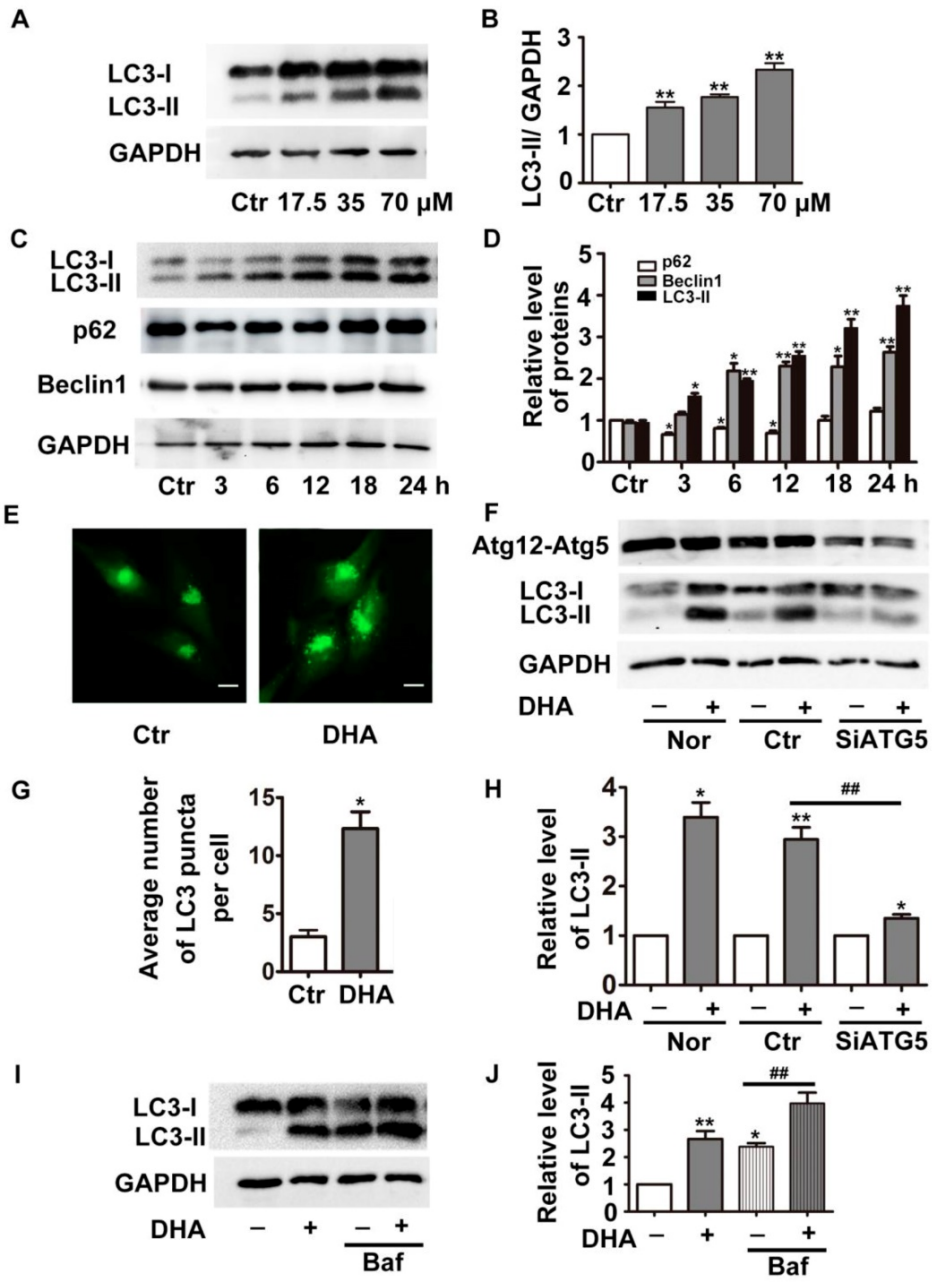
** DHA induces autophagy in HUVECs. (A)** HUVECs were treated with different doses of DHA for 24 h and the protein levels of LC3 were detected by Western blot.** (B)** Densitometry analysis of prtein bands in **(A)**. n=3, **p< 0.01. **(C)** HUVECs were treated with 35 μM DHA for different time and the protein levels of LC3 were detected by Western blot; **(D)** Densitometry analysis of protein bands in **(C)**, n=3; *p< 0.05, **p< 0.01. **(E, G)** Representative fluorescent images and quantifications of GFP-LC3 puncta in HUVECs. Magnification: 400×; Scale bar represents 10 μm. *p< 0.05. **(F, H)** HUVECs were transfected with scramble siRNA or siRNA targeting *ATG5* for 48 h, and were treated with 35 μM DHA for another 24 h. The knockdown efficacy of Atg5 and protein levels of LC3 were detected by Western blot. *p< 0.05, **p< 0.01, **^##^*** p*< 0.01.** (I, J)** Western blot analysis of LC3-II in HUVECs treated with 35 μM DHA for 6 h. Baf (100 nM) was added to the cells for 1 h prior to the DHA exposure. *p< 0.05, **p< 0.01, **^##^*** p*< 0.01.

During formation of autophagosomes, the autophagy-related protein LC3 is transformed from cytosolic form LC3-I to the membrane bound form LC3-II 16. The detection of LC3-I to LC3-II conversion is widely used to determine autophagy of cells 16. As shown in Fig. 1A-B, treatment with DHA (24 h) significantly increased the protein levels of LC3-II in a dose-dependent manner (*p*<0.01). DHA (35 μM) also gradually increased LC3-II protein level with prolongation of the treatment duration (Fig. 1C-D, *p*<0.05). The protein levels of p62 (a selective degradative substrate of autophagy) and beclin 1 (a critical autophagy-related protein) were also assessed. Fig. 1C-D showed that 35 μM DHA reduced protein levels of p62 for 3 h, 6 h and 12 h. Meanwhile, DHA significantly increased the protein levels of beclin 1. Then we transfected HUVECs with pcDNA3.1-GFP-LC3 plasmids, and observed increased GFP-LC3-positive punctuate structures in DHA-challenged cells, indicating increased formation of autophagosomes (Fig. 1E and G).

The elevation of LC3-II and the accumulation of autophagosomes may result from autophagy induction enhancement or autophagic flux blockade 16. To determine the function of DHA, we knockdown the critical autophagy initiating protein *ATG5* by siRNA transfection, which decreased ATG5 protein expression by more than 70% (Fig. 1F). If enhanced autophagy induction contributes to DHA-induced increase of LC3-II, the combination of DHA with *ATG5* siRNAs is supposed to induce a prominent decrease in LC3-II levels. Indeed, compared with scramble siRNA transfection, *ATG5* knockdown significantly abolished DHA-induced upregulation of LC3-II, suggesting that DHA induces autophagy in HUVECs. Furthermore, we assessed the level of LC3-II in the presence of bafilomycin A1 (Baf). Baf inhibits the fusion of autophagosome with lysosome through inhibiting the activity of vacuolar H^+^-ATPase, thereby blocks LC3-II degradation, leading to LC3-II accumulation 17. As shown in Fig. 1I-J, co-treatment with DHA and Baf (100 nM) in HUVECs induced a more significant accumulation of LC3-II than Baf alone, demonstrating that DHA indeed enhances autophagy.

### ROS production does not contribute to DHA-induced autophagy

**Figure 2:**
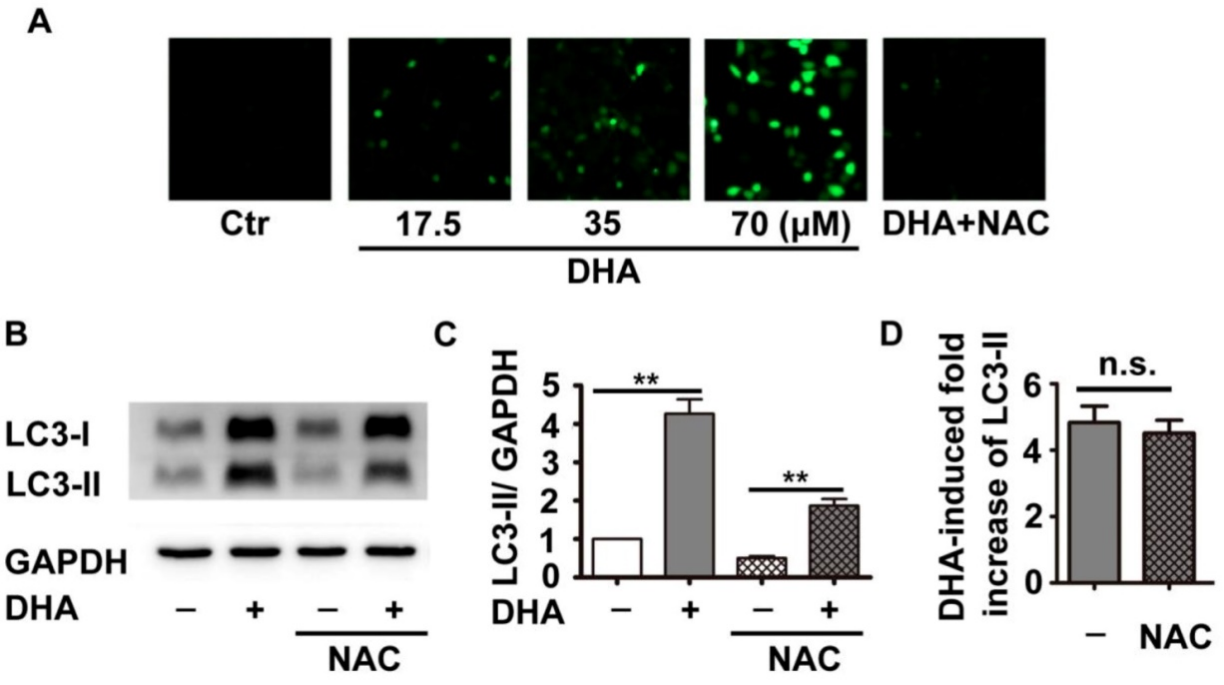
** Effects of DHA on ROS in HUVECs. (A)** HUVECs were treated with DHA at different concentrations in the absence or presence of the antioxidant NAC for 24 h. Intracellular ROS generation was detected by DCFH-DA staining with a fluorescence microscope. **(B)** After pretreatment of HUVECs with NAC (5 mM) for 1 h, DHA (35 µM) was added for another 24 h. Protein levels of LC3-II were detected by Western blot. **(C, D)** Densitometry analysis of protein bands in (B), n=3, **p< 0.01. n.s, not significant.

Previous studies have shown that DHA inhibits tumor growth partially through production of ROS 18. Inhibition of ROS reduces DHA-induced autophagy in cancer cells 19. Therefore, we investigated the role of ROS in DHA-induced autophagy in HUVECs. DCFH-DA, a fluorescent probe, was used to indicate cellular ROS levels. As shown in Fig. 2A, HUVECs treated with DHA (17.5, 35, 70 μM) showed a dose-dependent enhancement in fluorescence intensity compared to the solvent control group, suggesting that DHA increases ROS production. NAC is a usual anti-oxidant, and markedly suppressed DHA-induced ROS production (Fig. 2A). Although pretreatment with NAC significantly inhibited both basal and DHA-induced LC3-II protein levels (Fig. 2B-C, *p*<0.01), proportion of DHA-induced increase of LC3-II protein remained unchanged (Fig. 2D). Hence, DHA enhanced the production of ROS, which did not contribute to DHA-induced autophagy.

### MAPK does not mediate DHA-induced autophagy

**Figure 3:**
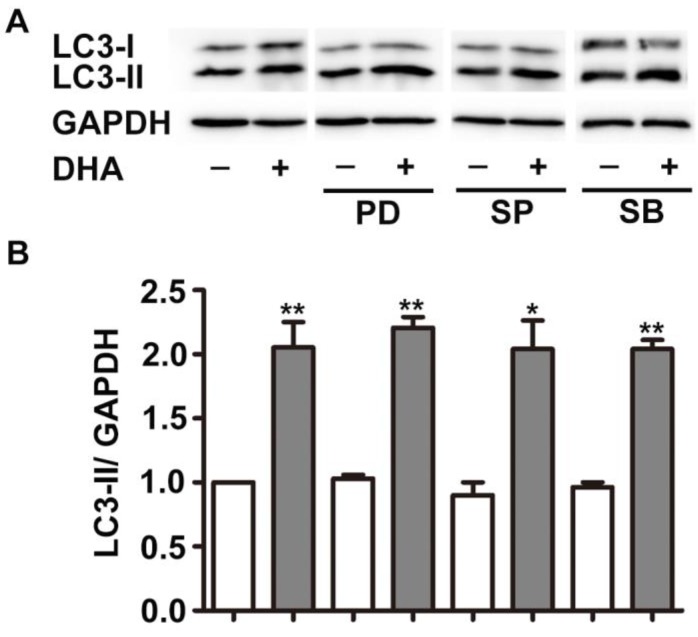
** MAPK signaling does not mediate DHA-induced autophagy in HUVECs.** HUVECs were treated with DHA for 24 h after pretreatment with ERK inhibitor PD98095 (PD), JNK inhibitor SP600125 (SP), or p38 inhibitor SB203580 (SB). The protein levels of LC3 were examined by Western blot. n=3, *p< 0.05, **p< 0.01.

MAPK cascades is another critical modulator of autophagy 20. The major members of MAPK, ERK, JNK, and p38 MAPK displayed specific regulatory patterns on autophagy 20, 21. Hence, we studied their roles in DHA-induced autophagy. As shown in Fig. 3, pretreatment with PD98059 (PD), SP6001225 (SP) and SB203580 (SB), inhibitor of ERK, JNK and p38 MAPK, did not alter the increment of LC3-II expression induced by DHA, suggesting that MAPK is not required in DHA-induced autophagy.

### DHA suppresses the activity of Akt/mTOR pathway in HUVECs

**Figure 4:**
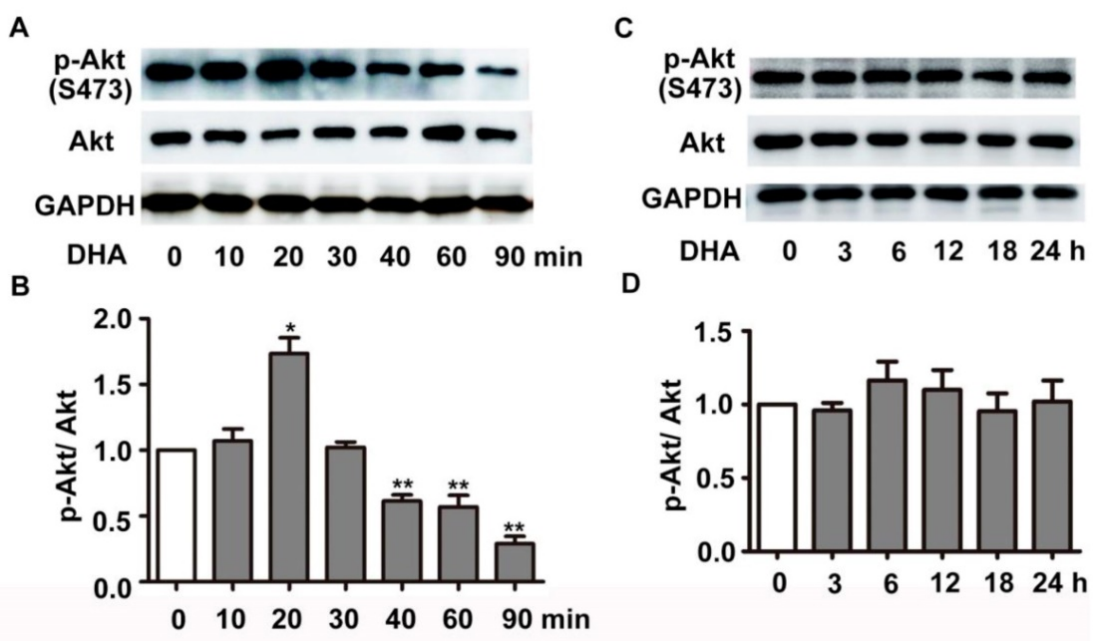
** Effects of DHA on Akt in HUVECs. (A-D)** HUVECs were treated with DHA for different time periods. Phosphorylated Akt and total Akt were detected by Western blot. n=3, *p< 0.05, **p< 0.01.

**Figure 5:**
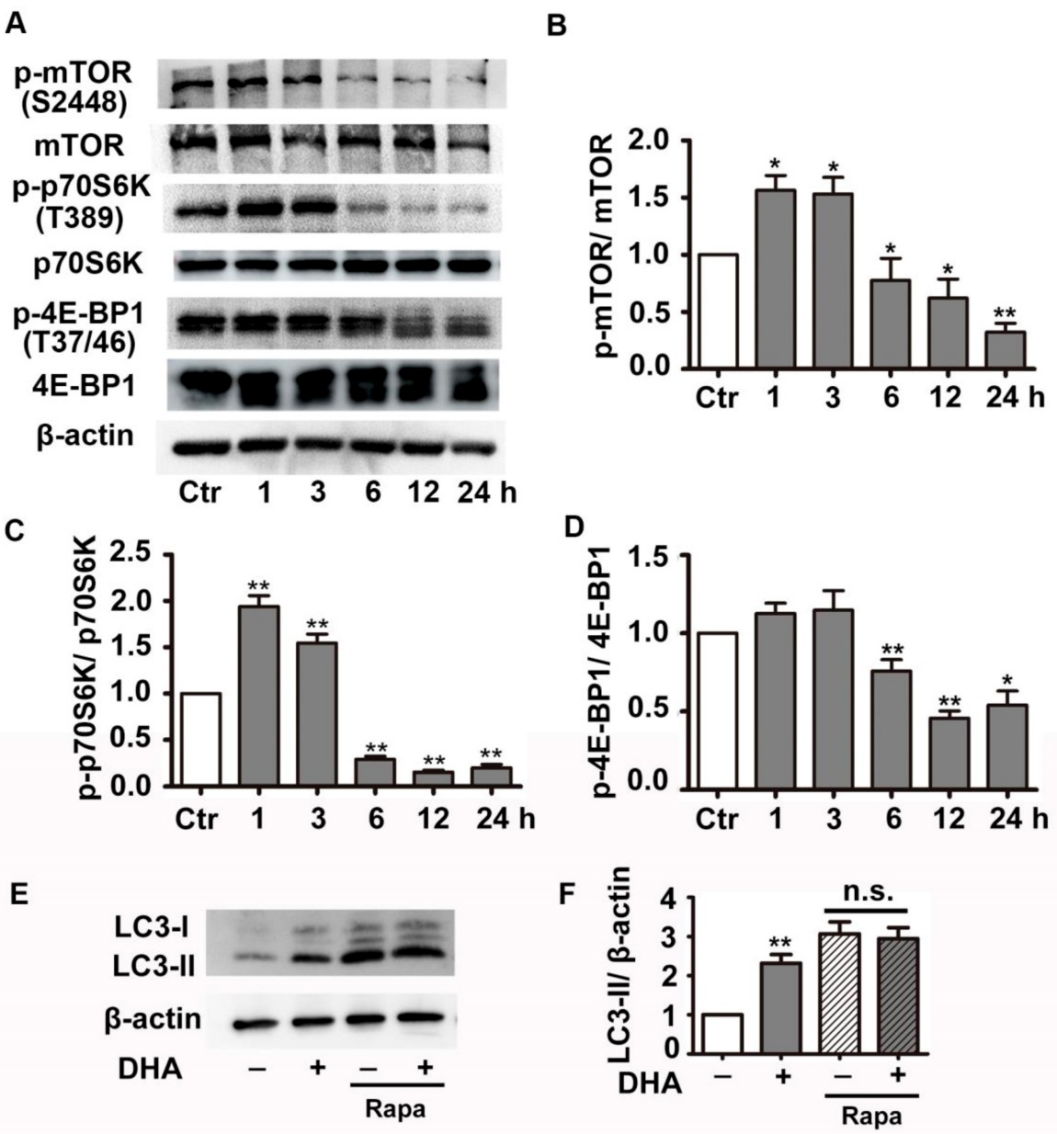
** Effects of DHA on mTOR signaling in HUVECs. (A-D)** Phosphorylation levels of mTOR, 4E-BP1, p70S6K in DHA-treated HUVECs were detected by Western blot. n=3, *p< 0.05, **p< 0.01. (E, F) HUVECs were pretreated with mTOR inhibitor (5 μM) and then treated with DHA for another 6 h. The protein levels of LC3 was determined by Western blot. n=3, **p< 0.01. n.s, not significant.

We next investigated the effect of DHA on PI3K/Akt/mTOR signaling pathway, a classical pathway regulating autophagy. HUVECs were treated with 35 μM DHA, and phosphorylation of Akt was measured by Western blot. Fig. 4A-B showed that phosphorylation of AKT (Ser473) increased at 20 min and was significantly inhibited by DHA treatment at 40 min or longer (*p*<0.01), suggesting a transient upregulation of Akt activity. However, Akt activity was inhibited by DHA from 40 min to 90 min. We also examined the phosphorylation of Akt after incubation of DHA for more than 1 hour since autophagy was induced by DHA at 3 h. Interestingly, incubation with DHA for a longer time did not affect phosphorylation of Akt (Fig. 4C-D). mTOR is a downstream substrate of the PI3K/AKT pathway 22. It is a critical Ser/Thr protein kinase that functions as an ATP and amino acid sensor to balance nutrient availability and cell growth 23. The downstream effectors of mTOR are mainly ribosomal p70S6 kinase protein (p70S6K) and eukaryotic initiation factor 4E binding protein 1 (4E-BP1) 23. Generally, mTOR negatively regulates autophagy though phosphorylating and inactivating ULK1/2 and ATG13 24. The expression of p-mTOR (Ser2448), p-p70S6K (Thr389), and p-4E-BP1 (Thr37/46) were detected in HUVECs after DHA treatment by Western blot. Fig. 5A-D showed that the protein levels of p-mTOR (Ser2448) and p-p70S6K (Thr389) in HUVECs increased at 1 h and 3 h. However, p-mTOR (Ser2448), p-p70S6K (Thr389) and p-4E-BP1 (Thr37/46) were decreased after 6 h treatment of DHA (*p*<0.05). The suppressed mTOR activity correlated with the occurrence of autophagy after 6 h, suggesting that mTOR may be involved in DHA-induced autophagy. Rapamycin (Rapa), an mTOR inhibitor, was used to validate the role of mTOR in DHA-induced autophagy. As shown in Fig. 5E-F, rapamycin (5 μM) alone significantly increased LC3-II protein levels, and DHA did not induce additional increase of LC3-II levels after pretreatment with rapamycin. These results suggested that mTOR mediates DHA-induced autophagy.

## Discussion

**Figure 6:**
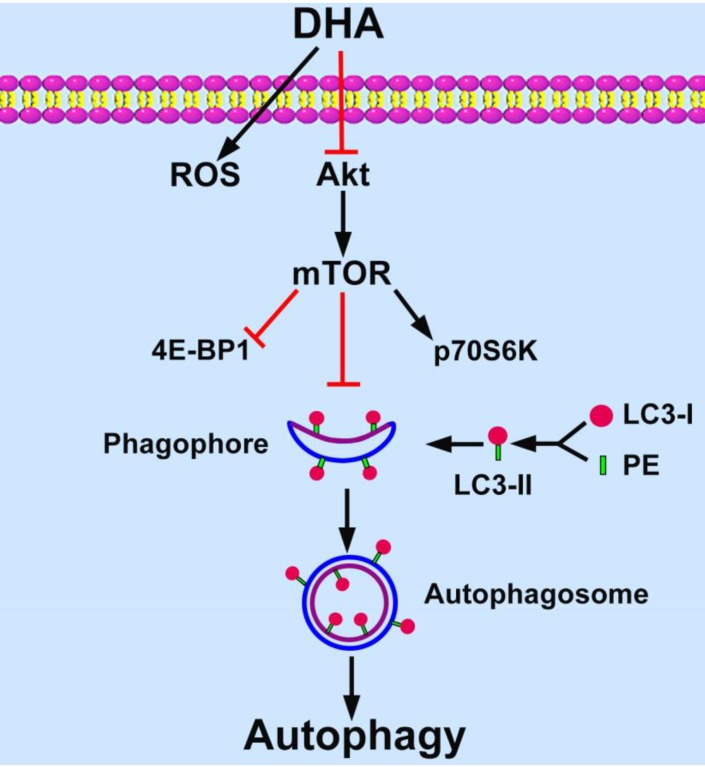
** Schematic diagram of proposed mechanisms of DHA-induced autophagy.** DHA suppresses Akt activity and its downstream mTOR signaling pathway. MTOR inhibits the formation of phagophore, which initiates autophagy by expanding to an autophagosome. During the expansion from phagophore to autophagosome, cytoplasmic LC3-I is conjugated with phosphatidyl ethanolamine (PE) and is changed into LC3-II. LC3-II is recruited to the membrane of phagophore and autophagosome, and then autophagy is induced.

Targeting angiogenesis becomes an important strategy for tumor treatment 3. DHA has been reported as an efficient anti-angiogenic agent in addition to its anti-malarial role 11, 12, 25. Recent studies show that angiogenesis relates closely with autophagy 9, 10. In the present study, we found that DHA induces autophagy in HUVECs. In addition, Akt/mTOR signaling pathway is inhibited by DHA and mediates DHA-induced autophagy (Fig. 6).

Artemisinin derivatives display cell-type specific effects on autophagy 19, 26-31. DHA induces autophagy in several cancer cell lines 26-28. In addition, DHA and artesunate promote autophagy in rat chondrocytes 29, 30. DHA also induces autophagy in activated hepatic stellate cells to facilitate its anti-inflammatory effect in liver fibrosis 19. However, artesunate inhibits autophagy to prevent cytokine release from macrophages 31. In our study, we first found that DHA significantly induces autophagy in endothelial cells, suggesting that autophagy may contribute to the anti-angiogenic effects of DHA.

It has been reported that the endoperoxy bridge of DHA breaks in the presence of ferrous ion, leading to the production of cytotoxic ROS, which is considered as a mechanism for the anti-tumor and anti-malarial effects of DHA 32. Accumulated evidence reveals that ROS produced under oxidative stress may induce autophagy to protect cells from oxidative stress associated damage 33. Our data show that DHA significantly increased intracellular ROS production in HUVECs. Pretreatment of antioxidant NAC reduces both basal and DHA-induced ROS levels. However, under the pretreatment of NAC, DHA still enhances the protein levels of LC3-II in a rate similar to that in NAC solvent control group. These observations suggest that NAC has no influence on DHA-induced autophagy.

MAPK family is a critical regulator of autophagy 20. DHA inhibits EC proliferation through suppression of ERK activity 15. In the current study, however, inhibition of ERK does not alter DHA induced autophagy. Jia *et al*. found that JNK activation mediates DHA-induced autophagy in pancreatic cancer cells 28. Zhang *et al*. also reported that activation of JNK is required for autophagy induction by DHA in hepatic stellate cells 19. In this study, inhibition of JNK has no effect on DHA-induced autophagy. Our previous study demonstrated that p38 MAPK is not required in DHA-induced suppression of EC migration 34. In this study, inhibition of p38 MAPK also has no effect on DHA-induced autophagy. These results suggest that DHA-induced autophagy in HUVECs is not mediated by MAPK pathway.

mTOR is a negative regulator of autophagy 24. It phosphorylates the critical autophagy protein ULK1 and inhibits its activity, which prevents formation of ULK1-Atg13-FIP200 complex, thereby, inhibits autophagy 24. In addition, mTOR signaling suppresses autophagy through phosphorylation of transcription factor EB and preventing its nuclear translocation for autophagy gene expression 35. However, autophagy may be induced independent of mTOR under certain circumstances 36, 37. Previous studies showed that DHA inhibits mTOR activity in rhabdomyosarcoma cells and non-small cell lung carcinoma cells 38, 39. In addition, DHA suppressed mTOR and induced autophagy in cisplatin-resistant ovarian cancer cells 27. Similarly, we demonstrated that DHA significantly inhibits Akt and mTOR activity in HUVECs. Rapamycin is an inhibitor of mTOR 40. The elevation of LC3-II by DHA is abolished by incubation with rapamycin, suggesting that mTOR mediates DHA-induced autophagy.

In conclusion, DHA induces autophagy of HUVECs in a dose- and time-dependent manner. Down-regulation of the activity of the Akt/mTOR pathway mediates DHA-induced autophagy. This study provides a better understanding of the mechanisms underlying anti-angiogenic properties of DHA.

## Abbreviations

ATG: autophagy-related protein
Akt: protein kinase B
DHA: dihydroartemisinin
DCFH-DA: dichlorofluorescein diacetate
GFP: green fluorescent protein
HUVEC: human umbilical vein endothelial cell
mTOR: mammalian target of rapamycin
NAC: N-acety-l-cysteine
NF-κB: nuclear factor-κB
4E-BP1: eukaryotic translation initiation factor 4E-binding protein 1
p70S6K: p70 ribosomal S6 kinase
ROS: reactive oxygen species
VEGFR: vascular endothelial growth factor receptor

## References

[1] Cross MJ, Claesson-Welsh L (2001). FGF and VEGF function in angiogenesis: signalling pathways, biological responses and therapeutic inhibition. Trends Pharmacol Sci.

[2] Wang Z, Dabrosin C, Yin X, Fuster MM, Arreola A, Rathmell WK (2015). Broad targeting of angiogenesis for cancer prevention and therapy. Semin Cancer Biol.

[3] DiPietro LA (2016). Angiogenesis and wound repair: when enough is enough. J Leukoc Biol.

[4] Yang WJ, Yang DD, Na S, Sandusky GE, Zhang Q, Zhao G (2005). Dicer is required for embryonic angiogenesis during mouse development. J Biol Chem.

[5] Wahl O, Oswald M, Tretzel L, Herres E, Arend J, Efferth T (2011). Inhibition of tumor angiogenesis by antibodies, synthetic small molecules and natural products. Curr Med Chem.

[6] Saha S, Panigrahi DP, Patil S, Bhutia SK (2018). Autophagy in health and disease: A comprehensive review. Biomed Pharmacother.

[7] Kroemer G, Marino G, Levine B (2010). Autophagy and the integrated stress response. Mol Cell.

[8] Virgin HW, Levine B (2009). Autophagy genes in immunity. Nat Immunol.

[9] Lu Q, Yao Y, Hu Z, Hu C, Song Q, Ye J (2016). Angiogenic factor AGGF1 activates autophagy with an essential role in therapeutic angiogenesis for heart disease. PLoS Biol.

[10] Li WD, Zhou DM, Sun LL, Xiao L, Liu Z, Zhou M (2018). LncRNA WTAPP1 promotes migration and angiogenesis of endothelial progenitor cells via MMP1 through microRNA 3120 and Akt/PI3K/autophagy pathways. Stem Cells.

[11] Chen HH, Zhou HJ, Fang X (2003). Inhibition of human cancer cell line growth and human umbilical vein endothelial cell angiogenesis by artemisinin derivatives *in vitro*. Pharmacol Res.

[12] Dong F, Zhou X, Li C, Yan S, Deng X, Cao Z (2014). Dihydroartemisinin targets VEGFR2 via the NF-kappaB pathway in endothelial cells to inhibit angiogenesis. Cancer Biol Ther.

[13] Dong F, Han J, Jing G, Chen X, Yan S, Yue L (2016). Dihydroartemisinin transiently activates the JNK/SAPK signaling pathway in endothelial cells. Oncol Lett.

[14] D'Alessandro S, Basilico N, Corbett Y, Scaccabarozzi D, Omodeo-Sale F, Saresella M (2011). Hypoxia modulates the effect of dihydroartemisinin on endothelial cells. Biochem Pharmacol.

[15] Dong F, Tian H, Yan S, Li L, Dong X, Wang F (2015). Dihydroartemisinin inhibits endothelial cell proliferation through the suppression of the ERK signaling pathway. Int J Mol Med.

[16] Klionsky DJ, Abdelmohsen K, Abe A, Abedin MJ, Abeliovich H, Acevedo Arozena A (2016). Guidelines for the use and interpretation of assays for monitoring autophagy (3rd edition). Autophagy.

[17] Yamamoto A, Tagawa Y, Yoshimori T, Moriyama Y, Masaki R, Tashiro Y (1998). Bafilomycin A1 prevents maturation of autophagic vacuoles by inhibiting fusion between autophagosomes and lysosomes in rat hepatoma cell line, H-4-II-E cells. Cell Struct Funct.

[18] Lu M, Sun L, Zhou J, Yang J (2014). Dihydroartemisinin induces apoptosis in colorectal cancer cells through the mitochondria-dependent pathway. Tumour Biol.

[19] Zhang Z, Guo M, Zhao S, Shao J, Zheng S (2016). ROS-JNK1/2-dependent activation of autophagy is required for the induction of anti-inflammatory effect of dihydroartemisinin in liver fibrosis. Free Radic Biol Med.

[20] Sui X, Kong N, Ye L, Han W, Zhou J, Zhang Q (2014). p38 and JNK MAPK pathways control the balance of apoptosis and autophagy in response to chemotherapeutic agents. Cancer Lett.

[21] Moriyama M, Moriyama H, Uda J, Kubo H, Nakajima Y, Goto A (2017). BNIP3 upregulation via stimulation of ERK and JNK activity is required for the protection of keratinocytes from UVB-induced apoptosis. Cell Death Dis.

[22] Heras-Sandoval D, Perez-Rojas JM, Hernandez-Damian J, Pedraza-Chaverri J (2014). The role of PI3K/AKT/mTOR pathway in the modulation of autophagy and the clearance of protein aggregates in neurodegeneration. Cell Signal.

[23] Laplante M, Sabatini DM (2012). mTOR signaling in growth control and disease. Cell.

[24] Kim J, Kundu M, Viollet B, Guan KL (2011). AMPK and mTOR regulate autophagy through direct phosphorylation of Ulk1. Nat Cell Biol.

[25] Wei T, Liu J (2017). Anti-angiogenic properties of artemisinin derivatives (Review). Int J Mol Med.

[26] Chen K, Shou LM, Lin F, Duan WM, Wu MY, Xie X (2014). Artesunate induces G2/M cell cycle arrest through autophagy induction in breast cancer cells. Anticancer Drugs.

[27] Feng X, Li L, Jiang H, Jiang K, Jin Y, Zheng J (2014). Dihydroartemisinin potentiates the anticancer effect of cisplatin via mTOR inhibition in cisplatin-resistant ovarian cancer cells: involvement of apoptosis and autophagy. Biochem Biophys Res Commun.

[28] Jia G, Kong R, Ma ZB, Han B, Wang YW, Pan SH (2014). The activation of c-Jun NH(2)-terminal kinase is required for dihydroartemisinin-induced autophagy in pancreatic cancer cells. J Exp Clin Cancer Res.

[29] Jiang LB, Meng DH, Lee SM, Liu SH, Xu QT, Wang Y (2016). Dihydroartemisinin inhibits catabolism in rat chondrocytes by activating autophagy via inhibition of the NF-kappaB pathway. Sci Rep.

[30] Feng FB, Qiu HY (2018). Effects of Artesunate on chondrocyte proliferation, apoptosis and autophagy through the PI3K/AKT/mTOR signaling pathway in rat models with rheumatoid arthritis. Biomed Pharmacother.

[31] Kuang M, Cen Y, Qin R, Shang S, Zhai Z, Liu C (2018). Artesunate attenuates pro-inflammatory cytokine release from macrophages by inhibiting TLR4-mediated autophagic activation via the TRAF6-beclin1-PI3KC3 pathway. Cell Physiol Biochem.

[32] Xu CC, Deng T, Fan ML, Lv WB, Liu JH, Yu BY (2016). Synthesis and *in vitro* antitumor evaluation of dihydroartemisinin-cinnamic acid ester derivatives. Eur J Med Chem.

[33] Azad MB, Chen Y, Gibson SB (2009). Regulation of autophagy by reactive oxygen species (ROS): implications for cancer progression and treatment. Antioxid Redox Signal.

[34] Guo L, Dong F, Hou Y, Cai W, Zhou X, Huang AL (2014). Dihydroartemisinin inhibits vascular endothelial growth factor-induced endothelial cell migration by a p38 mitogen-activated protein kinase-independent pathway. Exp Ther Med.

[35] Martina JA, Chen Y, Gucek M, Puertollano R (2012). MTORC1 functions as a transcriptional regulator of autophagy by preventing nuclear transport of TFEB. Autophagy.

[36] Lampada A, O'Prey J, Szabadkai G, Ryan KM, Hochhauser D, Salomoni P (2017). mTORC1-independent autophagy regulates receptor tyrosine kinase phosphorylation in colorectal cancer cells via an mTORC2-mediated mechanism. Cell Death Differ.

[37] Ying Z, Xia Q, Hao Z, Xu D, Wang M, Wang H (2016). TARDBP/TDP-43 regulates autophagy in both MTORC1-dependent and MTORC1-independent manners. Autophagy.

[38] Odaka Y, Xu B, Luo Y, Shen T, Shang C, Wu Y (2014). Dihydroartemisinin inhibits the mammalian target of rapamycin-mediated signaling pathways in tumor cells. Carcinogenesis.

[39] Mi YJ, Geng GJ, Zou ZZ, Gao J, Luo XY, Liu Y (2015). Dihydroartemisinin inhibits glucose uptake and cooperates with glycolysis inhibitor to induce apoptosis in non-small cell lung carcinoma cells. PLoS One.

[40] Edwards SR, Wandless TJ (2007). The rapamycin-binding domain of the protein kinase mammalian target of rapamycin is a destabilizing domain. J Biol Chem.

